# An *Arabidopsis* Transcriptional Regulatory Map Reveals Distinct Functional and Evolutionary Features of Novel Transcription Factors

**DOI:** 10.1093/molbev/msv058

**Published:** 2015-03-06

**Authors:** Jinpu Jin, Kun He, Xing Tang, Zhe Li, Le Lv, Yi Zhao, Jingchu Luo, Ge Gao

**Affiliations:** 1 State Key Laboratory of Protein and Plant Gene Research, College of Life Sciences, Center for Bioinformatics, Peking University, Beijing, P.R. China; 2 Monsanto Biotechnology R&D Center, Beijing, P.R. China; 3 State Key Laboratory of Systematic and Evolutionary Botany, Institute of Botany, Chinese Academy of Sciences, Beijing, P.R. China

**Keywords:** transcription factor, transcriptional regulation, network structure, novel family, wiring preference

## Abstract

Transcription factors (TFs) play key roles in both development and stress responses. By integrating into and rewiring original systems, novel TFs contribute significantly to the evolution of transcriptional regulatory networks. Here, we report a high-confidence transcriptional regulatory map covering 388 TFs from 47 families in *Arabidopsis*. Systematic analysis of this map revealed the architectural heterogeneity of developmental and stress response subnetworks and identified three types of novel network motifs that are absent from unicellular organisms and essential for multicellular development. Moreover, TFs of novel families that emerged during plant landing present higher binding specificities and are preferentially wired into developmental processes and these novel network motifs. Further unveiled connection between the binding specificity and wiring preference of TFs explains the wiring preferences of novel-family TFs. These results reveal distinct functional and evolutionary features of novel TFs, suggesting a plausible mechanism for their contribution to the evolution of multicellular organisms.

Novel genes can rapidly integrate into existing networks and effectively drive the evolution of phenotypes (Chen et al. 2010, 2013). By turning gene transcription on or off at a specific time in a given space, transcription factors (TFs) and transcriptional regulatory networks play key roles in both development and stress responses. After diverging from other kingdoms more than one billion years ago, plants have evolved a sophisticated and distinctive system to precisely regulate development and to rapidly respond to environmental changes. Particularly during plant landing, many novel TF families emerged, contributing to more complex morphogenesis and adaption to a dramatically changed environment (Lang et al. 2010; Zhang, Jin, et al. 2011; Jin et al. 2014), providing an intriguing case to investigate how novel TFs contribute to the evolution of transcriptional regulatory systems. However, the absence of a large-scale, high-quality transcriptional regulatory network in plants hinders the full understanding of the contribution of novel TFs to the evolution of transcriptional regulatory systems.

The identification of numerous validated regulatory interactions across the vast scientific literature concerning studies in *Arabidopsis*, the most widely used model plant, offers a unique opportunity to build a high-confidence transcriptional regulatory network (Aerts et al. 2008). In this study, we curated a literature-derived transcriptional regulatory map for *Arabidopsis* and revealed the heterogeneity of developmental and stress response subnetworks and the wiring preference of novel TFs in transcriptional regulatory systems. These results provide insight into the fate determination of novel TFs, suggesting a plausible mechanism for the contribution of novel TFs to the evolution of multicellular organisms.

## Results and Discussion

**Fig. 1.**
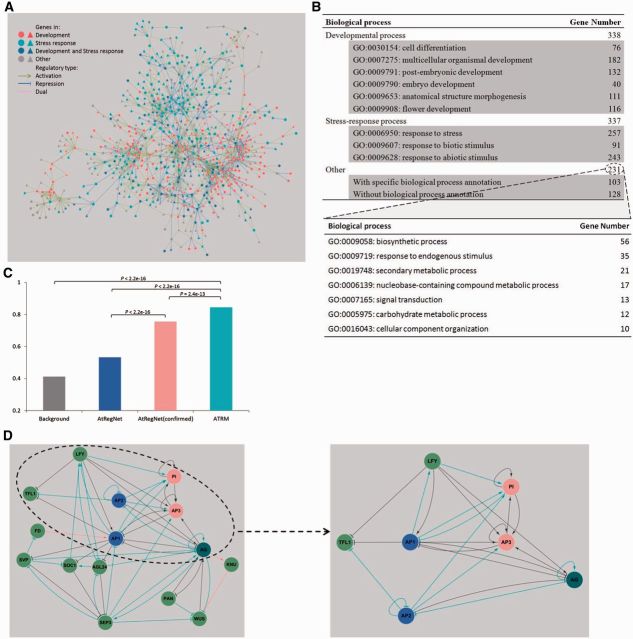
The transcriptional regulatory landscape in *Arabidopsis*. (*A*) The ATRM. This figure shows the largest connected component in the ATRM. The circle and triangle nodes represent TFs and non-TFs, respectively. (*B*) Biological process distribution of the genes in the ATRM. (*C*) The comparison of the proportion of regulations co-existing in the same biological processes indicates the high quality of the ATRM. The significance values from one-tailed binomial tests are indicated above the horizontal lines. (*D*) Comparison of the *Arabidopsis* floral meristem establishment and specification pathway summarized in a previously published review (Irish 2010) with the regulations among these genes established in the ATRM. The black line represents regulation present in both the summarized pathway and the ATRM; the red line represents regulation added to the ATRM after the comparison; and the cyan line represents novel regulation present in the ATRM but not observed in the summarized pathway. The blue, red, and cyan nodes represent the A, B, and C functional genes, respectively, in the classic “ABC” model of flower development (Weigel and Meyerowitz 1994).

Through binding specific *cis*-elements, TFs activate and/or repress the transcription of target genes. In recent decades, many transcriptional regulatory interactions between TFs and the promoters of their target genes have been experimentally determined either in vitro (e.g., by electrophoretic mobility shift assay and yeast one-hybrid) or in vivo (e.g., by chromatin immunoprecipitation). The regulatory activity (i.e., activation/repression) of these interactions can be further assessed through perturbations in the expression of the associated TFs. These functionally confirmed transcriptional regulatory interactions offer a unique opportunity to build a high-confidence *Arabidopsis* transcriptional regulatory network (Aerts et al. 2008). After the systematic literature mining and subsequent manual curation of each interaction through a review of the original texts, we constructed an *Arabidopsis* transcriptional regulatory map (ATRM) (fig. 1*A* and *B*, supplementary fig. S1 and Materials and Methods, Supplementary Material online). The current version of the ATRM covers 388 TFs from 81.0% (47 of 58) of the families in Plant Transcription Factor DataBase (PlantTFDB) (Zhang, Jin, et al. 2011), with direct supporting evidence from 974 peer-reviewed studies. The full data set is available online through an interactive web portal at http://atrm.cbi.pku.edu.cn/ (last accessed March 14, 2015).

TFs regulate the transcription of downstream targets and are involved in the same biological pathways as the target genes. The proportion of regulatory pairs co-occurring in the same biological process is typically used to evaluate the network quality (Wang et al. 2012). Based on gene ontology (GO) assignments, we determined that a significantly larger proportion of ATRM regulatory pairs were involved in the same biological processes than that of the highly reliable interactions in the AtRegNet confirmed data set (Yilmaz et al. 2011) (one-tailed binomial test, *P* = 2.4 × 10^−^^13^; fig. 1*C*), suggesting the high confidence of the ATRM. We then assessed the ATRM data set in the well-studied flower developmental process. In addition to successfully recalling 89% (24 of 27) of the known regulatory interactions in the reported pathway (Irish 2010), 27 novel regulatory interactions were further identified in the ATRM (fig. 1*D*). Interestingly, novel interactions for AP2 suggest that *AP2* might function as an “A class” gene in a manner similar to *AP1* by repressing *TFL1* to regulate the transition to the floral meristem (Bradley et al. 1997), activating the “B class” genes *AP3* and *PI* and mutually repressing each other via the “C class” gene *AG* (fig. 1*D*), consistent with findings that both *AP1* and *AP2* are required for A function (Bowman et al. 1991) (supplementary Materials and Methods, Supplementary Material online). Employing a Markov clustering algorithm, we further grouped genes in the ATRM into 156 densely, internally connected communities (Fortunato 2010). Among 62 communities with no less than 5 members, 93.5% (58 of 62) of the identified communities corresponded to specific biological processes (supplementary table S1, Supplementary Material online), demonstrating high-level cross-regulation among functional clusters in *Arabidopsis* (supplementary fig. S2, Supplementary Material online).

**Fig. 2.**
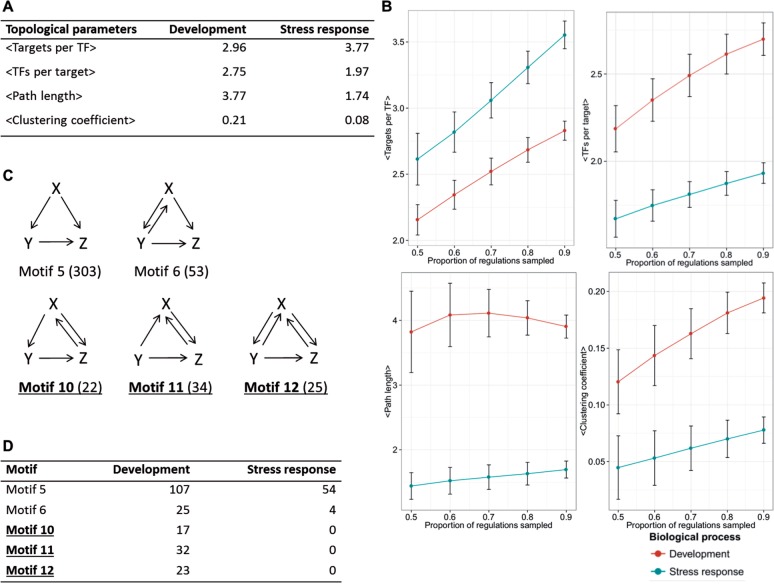
The architecture of developmental and stress response subnetworks in *Arabidopsis.* (*A*) Global topological parameters of the developmental and stress response subnetworks. (*B*) Topological parameters of the developmental and stress response subnetworks under subsamplings. We randomly sampled 50%, 60%, 70%, 80%, and 90% regulations from the developmental and stress response subnetworks 1,000 times and observed the effects on the calculated topological parameters. Standard deviations are indicated in the figure. (*C*) All identified three-node network motifs in the ATRM. The number in parentheses, for example, Motif 5 (303), represents the number of that motif present in the ATRM. (*D*) The distribution of network motifs in the developmental and stress response subnetworks. In panels (*C*) and (*D*), the network motifs absent from the unicellular organisms *Escherichia coli* and *Saccharomyces cerevisiae* are highlighted in bold (e.g., **Motif 10**).

On the basis of the GO annotation with experimental evidence, we identified the genes involved in developmental and stress response processes (fig. 1*B*). We further extracted interregulations among unambiguous developmental genes as developmental subnetworks, and the same for stress response subnetworks. Compared with the stress response subnetwork, the developmental subnetwork involved fewer targets per TF, more regulators per target, longer regulatory paths, and more interregulations among TFs (i.e., higher clustering coefficients) (fig. 2*A*). Follow-up analyses showed that the observed differences in global topological structures were robust and significant (fig. 2*B*, supplementary figs. S3–S5, Supplementary Data, and Materials and Methods, Supplementary Material online), consistent with previous observations between endogenous and exogenous subnetworks in yeast (Luscombe et al. 2004). Moreover, the binding specificities of TFs (measured by the information content of their binding matrices) involved in development were significantly higher than those of the TFs involved in the stress response (one-tailed Wilcoxon rank-sum test, *P* = 0.035; supplementary fig. S5, Supplementary Material online). These results showed that the developmental regulation is tighter and more complex than that of the stress response.

Transcriptional regulatory networks consist of a core set of network motifs (i.e., overrepresented regulatory patterns) (Lee et al. 2002; Milo et al. 2002; Shen-Orr et al. 2002; Gerstein et al. 2012; Neph et al. 2012; Boyle et al. 2014). Kinetic simulations and experimental studies have demonstrated that these motifs perform certain functions in transcriptional regulation (e.g., feed-forward loop for filtering noise and delaying the response when a signal is occurring or has ended; Mangan and Alon 2003) (Alon 2007). By systematically screening three-node regulatory patterns in the ATRM (supplementary fig. S6*A*, Supplementary Material online), we identified five three-node network motifs enriched in the ATRM (fig. 2*C*). Compared with the unicellular organisms *Escherichia coli* and *Saccharomyces cerevisiae*, there were three novel network motifs in the *Arabidopsis* transcriptional regulatory network (fig. 2*C* and supplementary table S3, Supplementary Material online). Compared with the motifs (motifs 5 and 6) enriched in *E. coli* and *S. cerevisiae*, the three novel motifs in *Arabidopsis thaliana* (motifs 10, 11, and 12) presented more complex regulations among TFs and were involved in developmental subnetworks, such as multicellular development, reproduction, and organ development (fig. 2*D* and supplementary table S4*A* and Supplementary Data, Supplementary Material online). Kinetic simulations confirmed the functionality of these three novel motifs in the maintenance and transition of gene expression states (supplementary fig. S6*B–D*, Supplementary Material online), which are critical for cell differentiation and fate decision in multicellular development (Alon 2007). Interestingly, one of the three novel motifs, motif 10, was not enriched in metazoan transcriptional regulatory networks (Gerstein et al. 2012; Neph et al. 2012; Boyle et al. 2014). Unlike animals, plants possess the capability for continuous organ regeneration during postembryonic development (Heidstra and Sabatini 2014).The kinetic simulation suggested that motif 10 could balance the maintenance and transition of developmental states (supplementary fig. S6*C*, Supplementary Material online), enabling it a potential role in the continuous organ regeneration of plants. Consistently, we observed that these motifs were preferentially involved in the maintenance and differentiation of meristems (supplementary table S4*C*, Supplementary Material online).

**Fig. 3.**
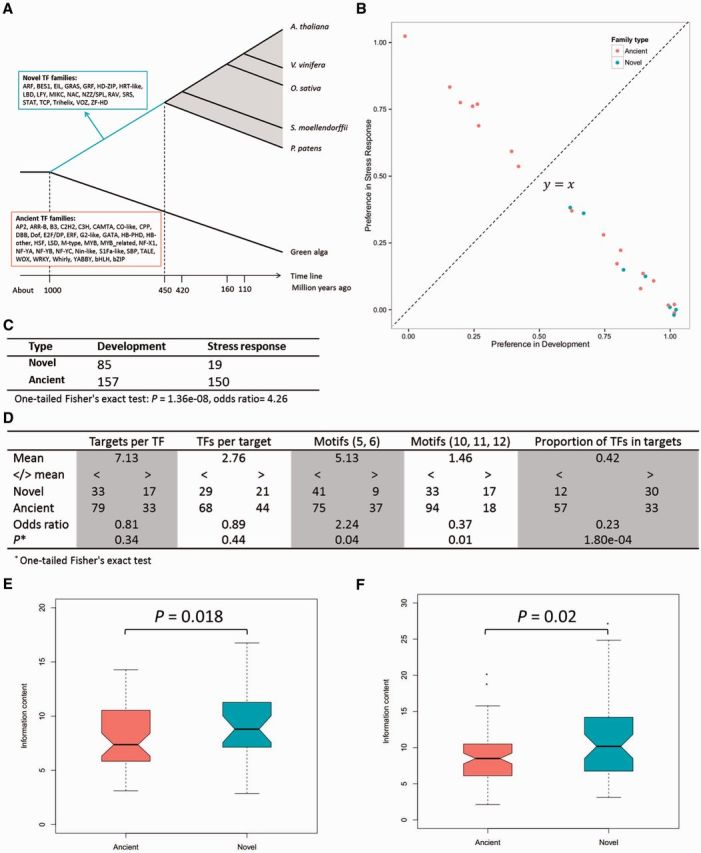
The wiring positions of ancient- and novel-family TFs in the *Arabidopsis* transcriptional regulatory system and their binding specificities. (*A*) The classification of ancient and novel TF families. Plant landing corresponds to ∼1 billion years ago to ∼450 Ma (cyan line). (*B*) The wiring preference of ancient and novel families in biological processes. Each point represents a family. A jitter function was used to finely modify the point positions to display overlapping points. (*C*) The distributions of ancient- and novel-family TFs in the developmental and stress response processes. (*D*) The wiring positions of ancient- and novel-family TFs in the ATRM. For each aspect, we summarized the numbers of novel- and ancient-family TFs that were fewer than or more than the average value. One-tailed Fisher’s exact tests were performed to compare the wiring preferences of novel- and ancient-family TFs. (*E* and *F*) The binding specificities of ancient- and novel-family TFs measured based on the information content of binding matrices in *Arabidopsis thaliana* (*E*) and *Homo sapiens* (*F*). In panels (*E*) and (*F*), the *P* values obtained from one-tailed Wilcoxon rank-sum tests are indicated above the horizontal line.

Individual TFs can be grouped into families based on their signature domains (primarily DNA-binding domains) (Riechmann et al. 2000; Zhang, Jin, et al. 2011). Novel TF families could emerge through innovations in novel signature domains or through new combinations of existing domains (Riechmann et al. 2000). Based on dating across 28 sequenced plants, we identified evolutionarily ancient TF families previously existing in green alga and novel TF families that emerged during plant landing (fig. 3*A* and supplementary tables S5 and Supplementary Data, Supplementary Material online). Compared with the TFs of ancient families, *Arabidopsis* TFs of novel families were preferentially wired into developmental processes (one-tailed Wilcoxon rank-sum test, *P* = 0.039 at the family level, and one-tailed Fisher’s exact test, *P* = 1.36 × 10^−8^ at the member level; fig. 3*B* and *C*). The GO enrichment analysis also indicated that these novel-family TFs were preferentially involved in developmental processes, particularly multicellular and organ development (supplementary table S6*B*, Supplementary Material online), confirming previous observations of the wiring bias of novel genes (Chen et al. 2010; Zhang, Landback, et al. 2011). In contrast, stress response processes were primarily enriched with TFs from ancient families (hyper-geometric test, *P* = 1.65 × 10^−69^; fig. 3*C*). By further investigating the wiring of these TFs in the transcriptional regulatory network, we determined that TFs of novel families preferred regulating TFs (one-tailed Fisher’s exact test, *P* = 1.80 × 10^−4^; fig. 3*D*) and were enriched in novel network motifs (motifs 10, 11, and 12, one-tailed Fisher’s exact test, *P* = 0.01; fig. 3*D*), whereas the TFs of ancient families were preferentially involved in network motifs already present in unicellular organisms (motifs 5 and 6, one-tailed Fisher’s exact test, *P* = 0.04; fig. 3*D*).

Previous studies have demonstrated that horizontally transferred TFs in bacteria are typically more tightly regulated to avoid detrimental effects (Rajewsky et al. 2002; Price et al. 2008; Perez and Groisman 2009), suggesting that novel TFs with less influence on the original system might have a greater probability of retention. When integrating into the original system, novel TFs with higher binding specificities tend to target fewer downstream genes, resulting in less “distortion” to existing circuits, and are less likely selected against than TFs with lower binding specificities. Compared with the TFs of ancient families, those of novel families did display higher binding specificities in *Arabidopsis* (one-tailed Wilcoxon rank-sum test, *P* = 0.018; fig. 3*E*), a finding that was also confirmed in *Homo sapiens* (one-tailed Wilcoxon rank-sum test, *P* = 0.02; fig. 3*F*).

Previous studies have shown that TFs of different hierarchical layers show significantly different properties (Jothi et al. 2009). Does the higher regulatory specificity of novel-family TFs contribute to their wiring preference? By comparing the information content of the corresponding binding matrices, we determined that TFs with higher binding specificities preferred regulating TFs rather than non-TFs (one-tailed Wilcoxon rank-sum test, *P* = 0.05; supplementary fig. S7*A*, Supplementary Material online) and tended to be wired into novel network motifs which presented more complex regulations among TFs (one-tailed Wilcoxon rank-sum test, *P* = 0.005; supplementary table S8*A*, Supplementary Material online). We further observed a significant correlation between the binding specificities of TFs and the proportion of TFs to their targets (Spearman’s rank correlation *ρ* = 0.46 and *P* = 0.02; supplementary table S8*B*, Supplementary Material online), which was also confirmed in bacteria *E. coli* (supplementary fig. S7*B* and Supplementary Data, Supplementary Material online), fungi *S. cerevisiae* (supplementary fig. S7*C* and Supplementary Data, Supplementary Material online), and metazoan *H. sapiens* (supplementary fig. S7*D* and Supplementary Data, Supplementary Material online), demonstrating a bona fide and generic connection between the binding specificities of TFs and their wiring preferences in transcriptional regulatory networks. Considering the rapid rewiring rate of transcriptional regulatory networks (Borneman et al. 2007; Schmidt et al. 2010; Shou et al. 2011) and the conservation of DNA-binding domains (Riechmann et al. 2000) and their binding specificities (Jolma et al. 2013; Weirauch et al. 2014), this connection might indicate that evolutionary selection plays a role in modeling the wiring positions of TFs. To a certain extent, the higher binding specificities of novel-family TFs might explain why novel-family TFs were preferentially wired into the novel motifs in the developmental processes of *A. thaliana.* We also inspected and excluded potential explanations, including the bias duplication of individual TFs, selective pressure for development during plant landing, and the wiring preference of young TF individuals (supplementary Materials and Methods, Supplementary Material online)*.*

Evolution from unicellular to multicellular organisms was a vital event in the evolution of life. Compared with the unicellular organisms *E. coli* and *S. cerevisiae*, the multicellular organisms *A. thaliana* and *H. sapiens* have more novel TF families (or novel-family TFs) in TF compositions (fig. 3*A* and supplementary table S7, Supplementary Material online) and more novel network motifs in transcriptional regulatory networks (fig. 2*C* and supplementary table S3, Supplementary Material online) (Gerstein et al. 2012; Neph et al. 2012). Compared with the ancient-family TFs, novel-family TFs typically have higher binding specificities (fig. 3*E* and *F*) and are preferentially wired into novel network motifs (fig. 3*D*) and more complex regulations among TFs in multicellular development (fig. 3*B* and *C* and supplementary table S6*B*, Supplementary Material online). These novel motifs can fulfill the functions required for multicellular development (supplementary fig. S6*B–D*, Supplementary Material online) (Alon 2007). These results highlight a role for novel-family TFs in multicellular development and indicate plausible roles for these TFs in the evolution of life toward multicellular organisms. Therefore, the results of this study not only provide a valuable resource to explore the *Arabidopsis* transcriptional regulatory system but also offer novel insight into the fate determination of novel TFs and their contributions to the evolution of transcriptional regulatory networks.

## Summary of Materials and Methods

*Arabidopsis* transcriptional regulatory interactions were collected using text-mining tools with further manual curation. Biological processes were assigned based on GO annotation with experimental evidence. The ATRM quality was evaluated by comparing the proportion of regulatory pairs co-existing in the same biological process. Global topological parameters of developmental and stress response subnetworks were calculated using igraph 0.6. Information contents of the binding matrices of TFs were calculated to demonstrate the binding specificities of these TFs. Network motifs were identified using Mfinder 1.2. Kinetic simulations of the function of novel network motifs were performed using ODE45. TF families were classified into ancient and novel families according to the time of emergence dated using 28 sequenced plants.

The full version of the “Materials and Methods” section and any associated references are available at *Molecular Biology and Evolution* online.

## Supplementary Material

Supplementary Data
