# Supplementary material for: An Arabidopsis Transcriptional Regulatory Map Reveals Distinct Functional and Evolutionary Features of Novel Transcription Factors
Source: Mol Biol Evol. 2015 Mar 6;32(7):1767–73. doi: 10.1093/molbev/msv058 (PMC4476157; doi:10.1093/molbev/msv058)
Supplement: Supplementary Data [file msv058_supplementary_data.zip › Supplementary_Material.pdf]

Supplementary Material

Materials and Methods

Literature mining and manual curation of transcriptional regulatory interactions in *Arabidopsis thaliana*

In this study, we used two data sources, PubMed Abstracts and ResNet Plant 3.0 (now called Pathway Studio Plant), a commercial knowledgebase for molecular relationships mined from more than 190,000 PubMed abstracts and 60,000 full-text articles from 28 plant-specific journals (for more details see http://www.elsevier.com/online-tools/pathway-studio/about/pathway-studio-plant) (Nikitin, et al. 2003), to collect verified transcriptional regulatory interactions in *A. thaliana*. Using each of the 1,701 *A. thaliana* transcription factors (TFs, 'proteins that show sequence-specific DNA binding and are capable of activating or/and repressing transcription') in PlantTFDB 2.0 (Zhang, et al. 2011) as input keywords, we retrieved 4,150 TF-associated interactions (supplementary fig. S1*A*) from ResNet Plant 3.0 and identified 3,211 TF-associated interactions from PubMed abstracts using MedScan (Novichkova, et al. 2003). After pooling the results, we obtained 4,663 TF-associated interactions.

We subsequently manually assessed each interaction in the following manner:

1) Screened for transcriptional regulatory interactions. Transcriptional regulatory interactions represent interactions between TFs and the promoters of the target genes. After reviewing the original texts, we removed 3,134 interactions falsely recognized by the text-mining tools or non-transcriptional regulatory interactions (*e.g.*, protein-protein interactions).
2) Identified transcriptional regulatory interactions missed by the text-mining tools. Through reviewing the original texts, we further identified 195 transcriptional regulatory interactions that were reported in original texts but missed by the text-mining tools.
3) Assigned a regulatory activity for each interaction. TFs activate and/or repress the transcription of target genes through binding specific *cis*-elements. Through reviewing original texts and original papers, we further assessed the regulatory activity (activation/repression) of each interaction and removed 55 interactions without determined regulatory activity.

4) Mapped the gene names of interactions to The *Arabidopsis* Information Resource (TAIR) IDs. To this end, we removed 26 interactions with gene names that could not be mapped to a unique TAIR ID and 212 redundant interactions.

Ultimately, we collected 1,431 functionally confirmed transcriptional regulatory interactions, 44.5% (637 of 1,431) of which represented regulations between two TFs in *Arabidopsis,* and constructed an *Arabidopsis* transcriptional regulatory map (ATRM) (supplementary fig. S1*B*).

Biological process assignment

Based on Gene Ontology (GO) annotation with experimental evidence (evidence code: EXP, IDA, IPI, IMP, IGI, or IEP; version: TAIR 6/05/2012) (Berardini, et al. 2004), we identified genes involved in developmental processes (GO:0032502 developmental process) and genes involved in stress response processes (GO:0006950 response to stress, GO:0009607 response to biotic stimulus, or GO:0009628 response to abiotic stimulus). The genes involved in both developmental and stress response processes were labeled "Dev. & Res." Genes lacking "biological process" annotations (including those without biological process annotation or with biological process annotation but without experimental evidence) and genes not involved in the developmental or stress response processes were classified as "other".

Quality evaluation of the ATRM

Because TFs regulate the transcription of target genes, TFs and target genes co-exist in the same biological process. The proportion of regulations that co-exist in the same biological process is typically larger in high-quality transcriptional regulatory networks than in low-quality networks. After combining all TFs ("TF list") in the regulator column of AtRegNet and the ATRM, we mapped their "biological process" (BP) annotations onto plant GO slim using the map2slim tool (http://search.cpan.org/~cmungall/go-perl/scripts/map2slim). We subsequently selected GO slim terms with no fewer than 10 mapped TFs and filtered out those terms too general for a TF, such as "regulation of RNA biosynthetic process," for continued analysis. TFs in the "TF list" and all genes mapped to the selected slim terms were marked as "mapped TFs" and "mapped genes", respectively. All combinations between "mapped TFs" and "mapped genes", except the self-regulations, were used as the background. The regulation in any of the selected biological processes described above was regarded as "co-existing" in the same process. One-tailed

binomial tests between different datasets were performed to confirm that the proportion of regulations in the same biological process of the test sample was no greater than that of the background. We evaluated the quality of the ATRM through comparisons of the proportion of regulations co-existing in the same biological process with the proportion of regulations in the background, AtRegNet, and AtRegNet (confirmed) (high-reliability regulations in AtRegNet (Yilmaz, et al. 2011)).

Identification of transcriptional regulatory communities

In networks, communities are defined as components with more intra-regulations than inter-regulations, which usually perform relatively specific functions (Fortunato 2010). Employing a Markov clustering algorithm, we classified the ATRM into 156 communities using CytoMCL 1.1 (Guzzi and Cannataro 2012) with an inflation parameter of 2.0. Sixty-two of the identified communities containing no fewer than five members were used for subsequent analyses. GO enrichment for each community was performed using topGO (Alexa and Rahnenfuhrer 2010), and genes with BP annotation were used as the background. The *P* values were adjusted for multiple tests using the method of Benjamini and Hochberg (Benjamini and Hochberg 1995). Based on the enriched GO terms, we assigned a name for the community whose enriched terms were consistent in terms of biological processes.

Measurement of the global topological structure

We used the largest connected component covering 98.7% and 94.2% of the regulations of the developmental and stress response sub-networks, respectively, as the representative network for the following analysis. Four parameters (<Targets per TF>, <TFs per target>, <Path length>, and <Clustering coefficient>; “< >” indicates the average value in networks) were used to measure the global topological structure of the transcriptional regulatory networks. In a transcriptional regulatory system, <Targets per TF> indicates how many targets could be immediately regulated by this TF; <TFs per target> measures the complexity with which a gene is regulated; <Path length> measures how long it takes for a signal to transfer from a TF to terminal genes; and <Clustering coefficient> indicates the complexity of regulations among TFs (Luscombe, et al. 2004). The global topological parameters of the representative developmental sub-network and stress response sub-network were calculated using igraph 0.6 (Csardi and Nepusz 2006).

Calculation of the information content of the TF binding matrices

High-quality TF binding matrices in plants were downloaded from TRANSFAC (professional 2011) (Matys, et al. 2006). The following methods were used to map the TRANSFAC id to the TAIR id. 1) For *A. thaliana* TFs with a gene alias in TRANSCFAC, we used the gene alias to map the TFs directly. 2) For TFs of other plant species or those without a gene alias in TRANSFAC, we used BLAST (Altschul, et al. 1997) to search against *Arabidopsis* genes to identify the best one-to-one pair. For TFs with two or more binding matrices, the matrix with the most sequences in construction was selected for the subsequent analysis.

The information content (IC) of the binding matrix of a TF measures the distinction of the binding profile from arbitrary sequences (Schneider, et al. 1986; Hertz and Stormo 1999). In this study, the previously described methods (Hertz and Stormo 1999) (equations 2 and 3) were adopted to calculate the IC of the binding matrices. To avoid any bias resulting from the difference in the sequence numbers used to construct the matrices, we adopted an adjusted pseudo count k in the calculation (equation 1). TFs with two or more binding matrices were used to determine k to ensure that the ICs of the TF binding matrices were comparable and not subject to systematic bias among matrices from different sequence numbers. AT1G13260 from the RAV family, which has two different types of DNA-binding domains, represented a special case, and we added the ICs of the two corresponding binding matrices together to represent its IC.

$$k = \begin{cases} 0.1s & if\ s \leq 10 \\ 1 + 0.02(s - 10) & if\ 10 < s\ \leq 20 \\ 1.2 + 0.005(s - 20) & if\ \ s\ > 20 \end{cases} \qquad (1)$$

$$f_{i,j} = \frac{n_{i,j} + p_i k}{\sum_{i=1}^{4} n_{i,j} + k} \qquad (2)$$

$$I = \sum_{j=1}^{w} \sum_{i=1}^{4} f_{i,j} \ln\left(\frac{f_{i,j}}{p_i}\right) \qquad (3)$$

k is the pseudo count added according to the number of sequences s used to construct this matrix; $n_{i,j}$ is the count of nucleotide i at position j in the binding matrix, p_i is the prior probability of nucleotide i, and $f_{i,j}$ is the corrected frequency of nucleotide i at position j; w is the width of the matrix, and I is the IC of the binding matrix.

To determine whether the calculated IC of the binding matrices of the TFs (Available at http://atrm.cbi.pku.edu.cn/download.php) could successfully represent the binding specificity of each TF, we predicted the putative target genes in the upstream 1,000 bp of the *A. thaliana* genome using Match (Matys, et al. 2006). A predefined cutoff for each matrix in TRANSFAC was used to minimize the sum of false positives and false negatives (Matys, et al. 2006). The high negative correlation between the IC of the binding matrix and the number of predicted targets (Spearman's rank correlation ρ = -0.76 and P = 7.72e-15) suggests that the IC of the binding matrices of TFs calculated using this method successfully represents the binding specificities of the TFs.

Identification of network motifs

We used Mfinder 1.2 (Milo, et al. 2002) to screen all possible three-node regulatory patterns (supplementary fig. S6*A*) and to identify enriched regulatory patterns among them. By generating 1,000 randomized networks with out-degree, in-degree, and mutual-degree conserved, we identified the three-node regulatory patterns that appeared significantly higher than those in randomized networks under default thresholds ($P < 0.01$, Mfactor > 1.10, and Uniqueness ≥ 4). P was calculated based on 1,000 randomized networks, Mfactor is the ratio between the number of this regulatory pattern in the real network and its number in randomized networks, and Uniqueness is the number of distinct sets of nodes involved in this regulatory pattern in the real network (Milo, et al. 2002).

To determine whether there was any novel network motif in the *Arabidopsis* transcriptional regulatory network compared with those of unicellular organisms, we also identified network motifs in *E. coli* and *S. cerevisiae*. The transcriptional regulatory network of *E. coli* was downloaded from RegulonDB 8.0 (Salgado, et al. 2013), and that of *S. cerevisiae* with direct evidence and confirmed function was retrieved from YEASTRACT (Abdulrehman, et al. 2011). We further classified the network motifs in the ATRM into two classes, motifs in development and motifs in stress responses, based on the following criteria: when two or more nodes of the three nodes were involved in the developmental process and no node was involved in the stress response process, this motif was assigned as a motif in development, and vice versa for motifs in the stress response. The TFs with BP annotations were used as the background for the biological

process enrichment analysis of the TFs involved in network motifs. *P* was adjusted for multiple tests using the method of Benjamini and Hochberg (Benjamini and Hochberg 1995).

Kinetic simulation of the novel network motifs in *Arabidopsis*

The changing rate of the transcription level of a gene represents the combined effect of the basic transcription rate, the rate of transcriptional activation/repression of other genes, and the degradation rate. We referred to kinetic equations of a previous study (see equations 4-6) (Mangan and Alon 2003) to simulate the function of the novel network motifs in *Arabidopsis*. In equations 4-6, B_i represents the basic transcription rate of gene *i*. K_{ji} is the transcriptional activation or repression coefficient of gene *i* through gene *j*, and $f\left(j, K_{ji}\right)$ is the transcriptional activation/repression rate of gene *i* through gene *j*. For activation, $f(j, K_{ji}) = K_{ji}C_j/(1+ K_{ji}C_j)$, and for repression, $f(j, K_{ji}) = (1-K_{ji}C_j)/(1+ KC_{max})$, where C_j is the transcription level of gene *j*. β_i is the degradation rate of gene *i*. For a gene activated through two TFs, *e.g.*, Z activated through X and Y, the equation $f(X, K_{xz}, Y, K_{yz}) = (K_{xz}C_x + K_{yz}C_y)/(1 + K_{xz}C_x + K_{yz}C_y)$.

$$dX/dt = B_x + \alpha_x f\left(Y, K_{yx}, Z, K_{zx}\right) - \beta_x X \qquad (4)$$

$$dY/dt = B_y + \alpha_y f\left(X, K_{xy}, Z, K_{zy}\right) - \beta_y Y \qquad (5)$$

$$dZ/dt = B_z + \alpha_z f\left(X, K_{xz}, Y, K_{yz}\right) - \beta_z Z \qquad (6)$$

In our simulation, the lowest transcription level of gene *i* was 0, and the highest level was 1, with a maximum allowable activation/repression rate of 1 ($\sum KC \leq 1$). To fulfill this constraint, we used $B_i = 0$, $\alpha = 1$, $\beta = 0.5$, and $K_{ji} = 1$. The high expression of gene X represented the signal occurring during a defined period (supplementary fig. S6*B*), and X was transcribed with the highest rate 1 at the beginning (supplementary figs. S6*C* and S6*D*). Genes Y and Z were initially transcribed with rate 0. When the transcription levels of Y and Z were no less than 0.5, these genes were activated to activate/repress the transcription of related genes. The high expression of X represented one state, and the high expression of Z represented another state. The kinetic simulations were performed using ODE45 in MATLAB. The MATLAB source codes are available at http://atrm.cbi.pku.edu.cn/download.php.

Classification of ancient and novel TF families

TFs are classified into 58 families according to their signature domains in PlantTFDB 2.0 (Zhang, et al. 2011). By dating their birth times based on 28 plants with sequenced genomes, we classified 54 TF families appearing in the most recent common ancestor (MRCA) of land plants into two types: ancient and novel families. TF families present in any of the nine green alga species were defined as ancient families, and TF families present in the MRCA of 19 land plants, but absent from the nine green alga species, were defined as novel families.

To determine whether the ancient families were previously present in *E. coli, S. cerevisiae,* or *H. sapiens,* we used the built-in TF prediction pipeline of PlantTFDB 2.0 (Zhang, et al. 2011) to scan the genome proteins of the three species under a relaxed cutoff (e-value ≤ 0.01 for sequence cutoff and domain cutoff). Based on the presence in *E. coli*, *S. cerevisiae*, or *H. sapiens*, ancient families were further divided into two types, "Ancient1" and "Anicent2". When ancient families were identified in any of the three species, these families were classified as "Ancient1"; otherwise, these families were classified as "Ancient2".

The wiring preference of ancient and novel TF families in biological processes

We used a "preference index," the proportion of genes involved in the developmental or stress response processes, to represent the wiring preference of TF families in biological processes. TFs involved in the developmental or stress response processes were clustered using BLASTClust (Altschul, et al. 1997) to merge highly redundant sequences (cutoff: coverage 0.9 and identity 0.9). Each cluster was regarded as a "Refgene" in this study. Refgenes only in developmental or stress response processes and families that included no fewer than five Refgenes were used to calculate the preference index.

The wiring positions of ancient- and novel-family TFs in the ATRM

We compared the following aspects of the wiring of ancient- and novel-family TFs: "Targets per TF," "TFs per target," the number of Motifs (5, 6) involved, the number of Motifs (10, 11, 12) involved, and the proportion of TFs to target genes. The first two aspects were used to determine whether there was any bias in the connectivity between the TFs of ancient and novel families; the last three aspects were used to determine whether there was any wiring preference in the transcriptional regulatory network between the TFs of ancient and novel families. Self-regulations in the ATRM were removed in this analysis. TFs in the ATRM with a degree of no

fewer than four (the median degree of TFs in the ATRM) were used to calculate the wiring of these TFs in the network. When calculating the proportion of TFs in the targets, we only used TFs with no fewer than four targets. For each aspect, we summarized the numbers of novel- and ancient-family TFs that were fewer than and more than the average value. One-tailed Fisher's exact tests were performed to compare the wiring preferences of novel- and ancient-family TFs.

Classification of ancient and novel TF families in *E. coli, S. cerevisiae*, and *H. sapiens*

The TFs of *E. coli*, *S. cerevisiae*, and *H. sapiens* were downloaded from DBD (Wilson, et al. 2008), and only TFs predicted through Pfam HMMs were used in this study. The taxonomic distribution of TF families was adopted from V. Charoensawan *et al.* (Charoensawan, et al. 2010). Novel families in *E. coli*, *S. cerevisiae,* and *H. sapiens* were considered as those with taxonomic distributions limited to Proteobacteria, Fungi, and Metazoa, respectively.

Binding specificities of TFs and the proportion of TFs to target genes in *E. coli, S. cerevisiae*, and *H. sapiens*

The transcriptional regulatory networks in *E. coli*, *S. cerevisiae*, and *H. sapiens* were downloaded from RegulonDB 8.0 (Salgado, et al. 2013), YEASTRACT (Abdulrehman, et al. 2011), and the ENCODE project (Gerstein, et al. 2012), respectively. The TF binding matrices of *E. coli* were downloaded from RegulonDB 8.0 and those of *H. sapiens* were downloaded from TRANSFAC (professional 2011, only matrices from SELEX were used) (Matys, et al. 2006). For *E. coli* and *H. sapiens*, the ICs of the TF binding matrices were used to represent the binding specificities of TFs. Owing to the low resolution of the TF binding matrices and the rich chromatin immunoprecipitation (ChIP)-chip data for *S. cerevisiae*, we directly used target numbers to represent the binding specificities of TFs with genome-wide ChIP-chip experiments, and the proportion of TFs to target genes indicated the proportion of TFs in the functionally confirmed regulations for *S. cerevisiae*. The classification of TFs and non-TFs in these three species was based on the following GO annotations: Ecocyc & EcoliHub (version: 3/15/2013) for *E. coli*, SDG (version: 4/43/2013) for *S. cerevisiae,* and EBI (version: 4/15/2013) for *H. sapiens*.

Clustering of TFs descended from a common ancestor

To investigate whether the wiring preference of novel-family TFs resulted from bias duplication of TF individuals, we downloaded orthologous genes to TFs of *A. thaliana* in *A. lyrata, V. vinifera, O. sativa*, and *P. patens* from Ensembl Plants (Release 15) (Kersey, et al. 2012), respectively. These data were used to cluster TFs that descended from the common ancestors at four key time points in the evolution of *A. thaliana* as “Refgenes” to investigate the distribution of these TFs in biological processes (supplementary fig. S8). Only TFs with orthologous genes in these species were used in this study.

Identification of TF individuals born during plant landing and classification of old and young TF individuals

Using the methods described above, we clustered the TFs of *A. thaliana* into 123 Refgenes in the MRCA of *A. thaliana* and *P. patens* (supplementary fig. S8). According to the orthologous assignment of Ensembl Plants (Release 15), we further assigned Refgenes without orthologous genes in *C. merolae* and *C. reinhardtii* as genes born during plant landing. For Refgenes with the same orthologous gene(s) in *C. reinhardtii,* the Refgene with the highest sequence similarity to its orthologous gene(s) was assigned as the gene born before this period, and other Refgene(s) in this orthologous group were assigned as genes born during this period.

Using the same method, we assigned *A. thaliana* TF individuals in the 123 Refgenes born before and after the divergence of the MRCA of *A. thaliana* and *P. patens* as old and young TF individuals, respectively.

Supplementary Text:

Comparison of the regulations in the ATRM with the reported *Arabidopsis* floral meristem establishment and specification pathway

By allowing us to integrate transcriptional regulatory interactions reported in the vast literature, literature mining effectively unveiled "novel" interactions that would be "invisible" when considering any single source, facilitating the identification of novel global patterns and the generation of new hypotheses. Irish previously summarized the *Arabidopsis* floral meristem establishment and specification pathway (Irish 2010). When retrieving interactions among these genes in the reported pathway from the ATRM, 89% (24 of 27) of the known regulations in the reported pathway were successfully recalled. In addition, the ATRM added another 27 novel edges to this reported pathway (fig. 1*D*). Interestingly, novel interactions for AP2 revealed a potential mechanism for the function of *AP2* as an A-class gene.

Robustness and significance of the differences in the global topological structures of the developmental and stress response sub-networks

We observed that the developmental and stress response sub-networks were different in global topological structures. Owing to the limitations of the current studies, the ATRM does not incorporate all of the transcriptional regulations in *Arabidopsis*. To determine whether the observed differences reflected a connectivity bias arising from the collection of regulations, we compared the connectivity of TFs involved in developmental and stress response processes, and observed no significant difference in either the ATRM (two-tailed Wilcoxon rank-sum test P = 0.71; supplementary fig. S3*A*) or the largest connected networks used to calculate the global topological parameters (two-tailed Wilcoxon rank-sum test P = 0.93; supplementary fig. S3*B*).

We further performed the following analyses to assess the robustness and significance of the observed differences. To determine whether the representative sub-networks sampled from the *Arabidopsis* transcriptional regulatory networks robustly reflected the differences between the two sub-systems, we randomly sampled 50%, 60%, 70%, 80%, and 90% of the regulations from the developmental and stress response sub-networks 1,000 times, and the results showed that the differences between these two sub-networks were robust (fig. 2*B*). To determine whether the observed differences reflected the different sizes of the developmental and stress response sub-networks, we sampled developmental sub-networks comprising the same number of regulations

as those in the stress response sub-networks 10,000 times, which revealed that the differences between these sub-networks were significant (supplementary fig. S4*A*). In addition, we obtained consistent results using a different version of the GO annotation (supplementary fig. S4*B*) or with genes involved in both developmental and stress response processes counted during network classification (supplementary fig. S4*C*). Moreover, the binding specificities of the TFs and the predicted networks in *A. thaliana* generated consistent results (supplementary fig. S5 and supplementary table S2).

These results demonstrate that the differences in the global topological structures of the developmental and stress response sub-networks are robust and significant.

Other possible reasons for the wiring preference of novel-family TFs in biological processes

Why are TFs of novel families preferentially wired into developmental processes rather than stress response processes? Potential explanations include the following: 1) bias duplication of some TF individuals; 2) selective pressure for development during plant landing; 3) the wiring preferences of young TF individuals; or 4) the properties of novel-family TFs. Discussions of explanations 1-3 follow:

1) Does the wiring preference reflect the bias duplication of some TF individuals?

Clustering TFs descended from the common ancestors as “Refgenes” at four key time points in the evolution of *A. thaliana* yielded consistent results (supplementary table S9), suggesting that the observed wiring preference was persistently present in its evolutionary history and cannot be explained as the bias duplication of some TF individuals.

2) Did the wiring preference result from selective pressure for development during plant landing?

By dividing ancient families into families present or absent in *E. coli*, *S. cerevisiae*, or *H. sapiens*, we determined that later-born ancient families were also preferentially wired into developmental processes (supplementary fig. S9). Compared with the ancient-family TFs born during plant landing, TFs of novel families still showed the same wiring preference (supplementary table S10), demonstrating that the observed preference did not reflect the potential selective pressure for development during certain periods.

3) Did the wiring preference result from the wiring preferences of later-born TF individuals?

A comparison of the wiring positions of TF individuals born during different periods revealed that later-born TF individuals did not display wiring preferences for developmental processes

(supplementary table S11), confirming that the properties of novel-family TFs, and not their time of birth, affected the wiring preferences of the novel-family TFs.

Supplementary Figures:

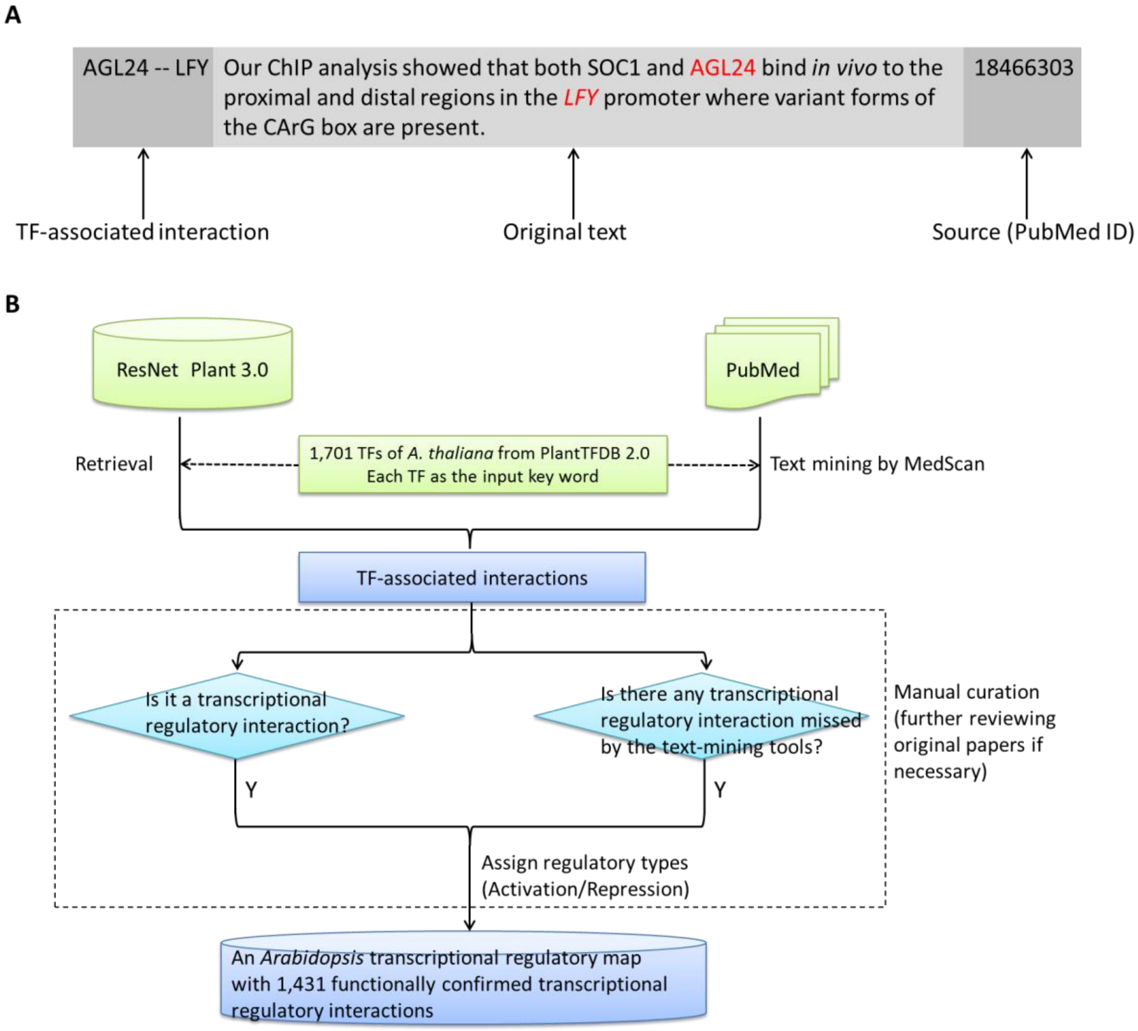

Supplementary Fig. S1. (*A*) An example of TF-associated interactions retrieved from ResNet Plant 3.0 or mined from PubMed Abstracts using MedScan. (*B*) The pipeline for the literature mining and manual curation of transcriptional regulatory interactions in *A. thaliana*.

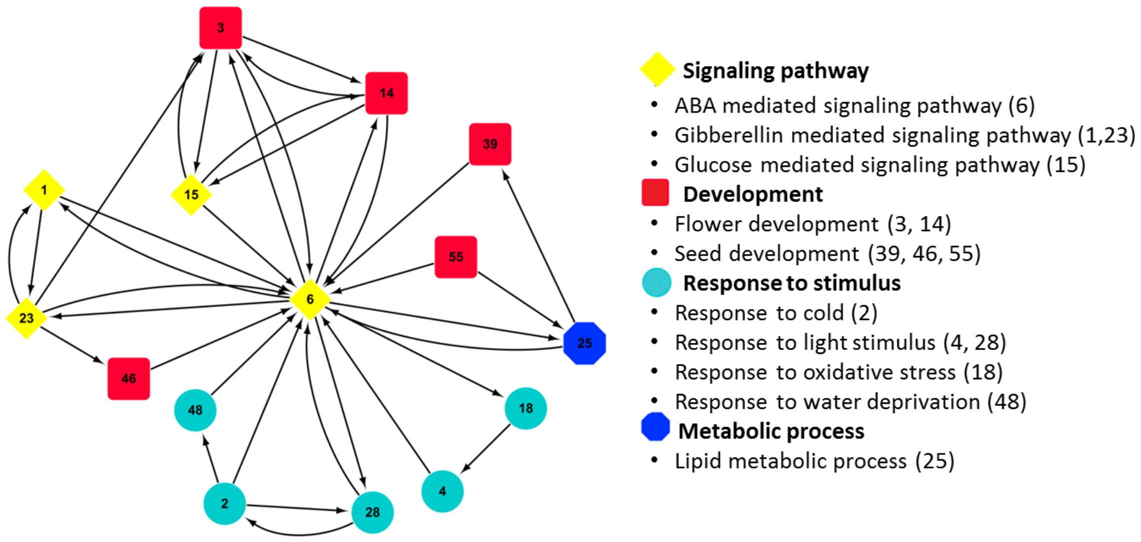

Supplementary Fig. S2. Cross-regulation with the abscisic acid (ABA)-mediated signaling pathway among communities in the ATRM. ABA plays important roles in regulating multiple developmental and stress response processes (Cutler, et al. 2010). The ATRM reveals the corresponding cross-regulation of the ABA-mediated signaling pathway with the gibberellin- and glucose-mediated signaling pathways, the development of seeds and flowers, and the types of stresses assessed at the transcriptional level. The numbers in parentheses correspond to community IDs. The arrows represent the direction of regulation between the genes in the communities.

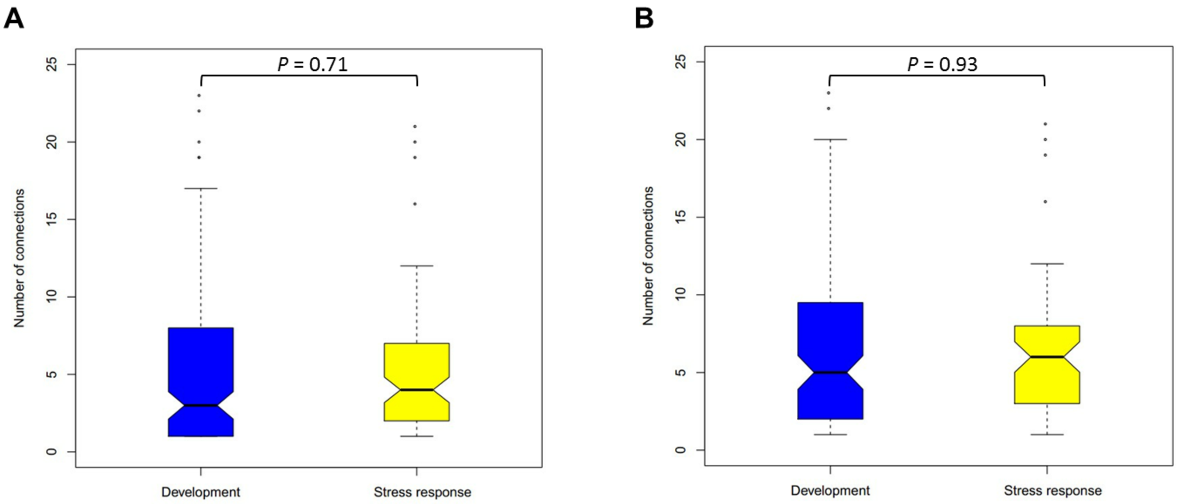

Supplementary Fig. S3. Connectivity of the TFs involved in developmental and stress response processes in the ATRM. (*A*) The number of collected connections for the TFs in the ATRM. (*B*) The number of collected connections for the TFs in the largest connected component used to calculate the global topological parameters. Two-tailed Wilcoxon rank-sum tests were performed to compare the connectivity of the TFs in the developmental process and the TFs in the stress response process.

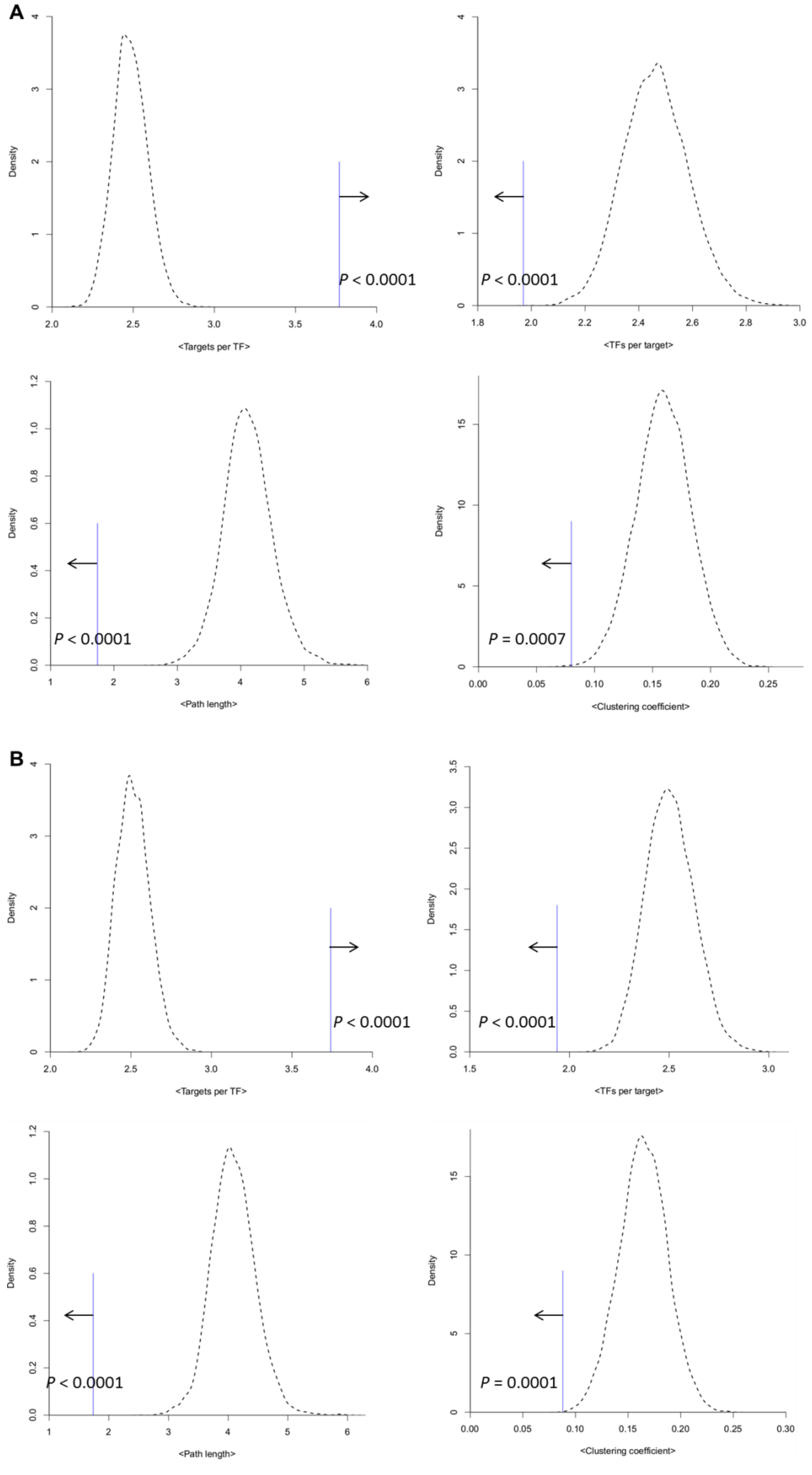
A
Density
P < 0.0001
<Targets per TF>
P < 0.0001
<TFs per target>
P < 0.0001
<Path length>
P = 0.0007
<Clustering coefficient>
B
P < 0.0001
<Targets per TF>
P < 0.0001
<TFs per target>
P < 0.0001
<Path length>
P = 0.0001
<Clustering coefficient>

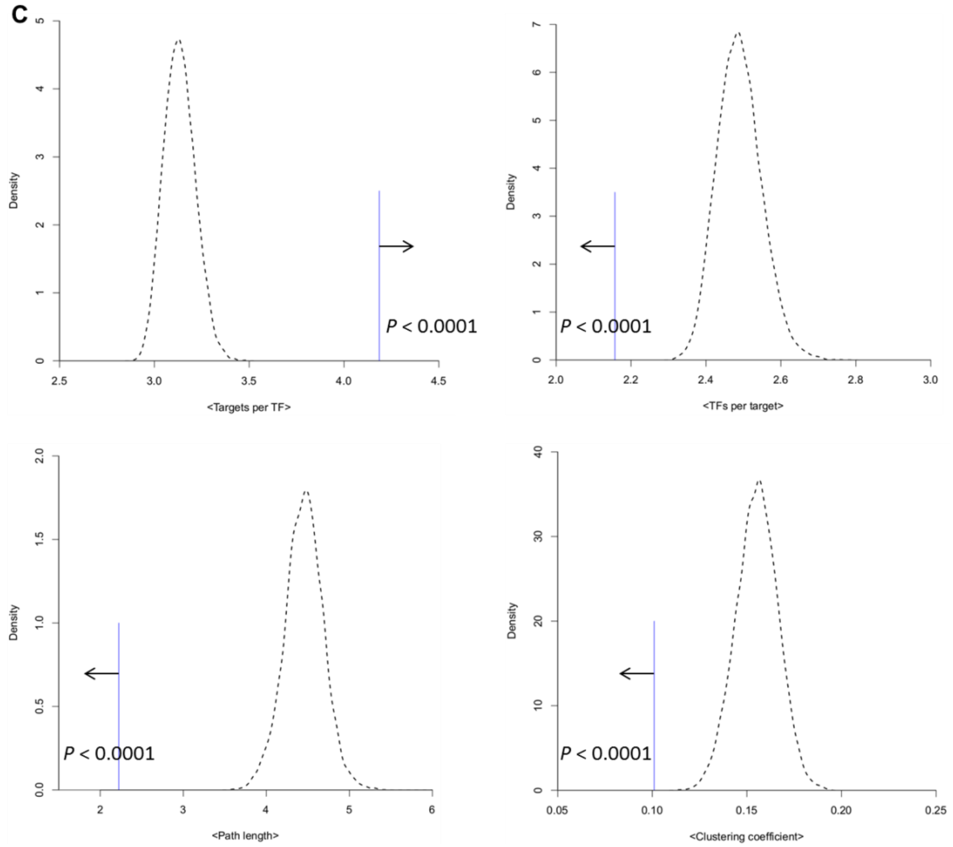

Supplementary Fig. S4. Significance and robustness of the differences in the global topological structures of developmental and stress response sub-networks. (*A*) Comparison of the global topological structures of developmental and stress response sub-networks. (*B*) Comparison of the global topological structures of developmental and stress response sub-networks using a different Gene Ontology (GO) annotation (combined GO annotations from TAIR10 and EBI, version 4/09/2013). (*C*) Comparison of the global topological structures of developmental and stress response sub-networks with genes involved in both developmental and stress response processes counted during sub-network classification. We sampled 10,000 times from the developmental sub-network using the same edge size as that in the stress response sub-network. The black dashed lines show the density distribution of the global topological parameters of the sampled developmental sub-networks, and the blue lines show the corresponding values for the stress response sub-networks. The *P* values were calculated based on 10,000 samples.

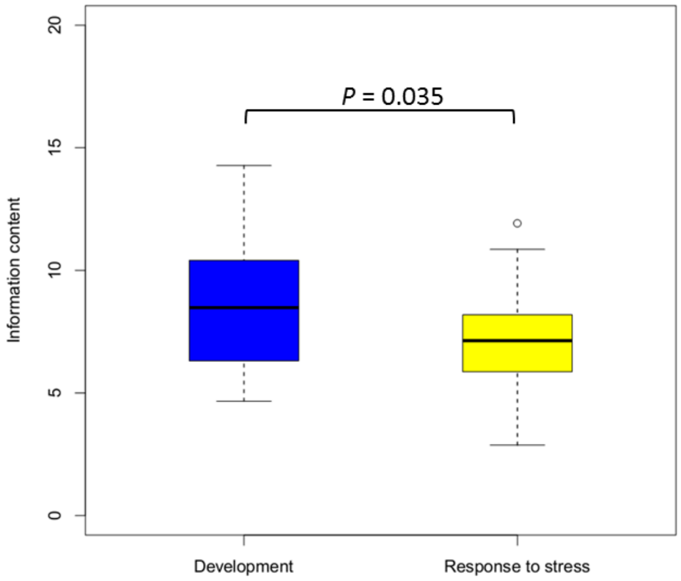

Supplementary Fig. S5. Binding specificities of TFs involved in the developmental and stress response processes measured based on the information content (IC) of the binding matrices. A one-tailed Wilcoxon rank-sum test was performed to compare the ICs of the TFs in the developmental process and the TFs in the stress response process.

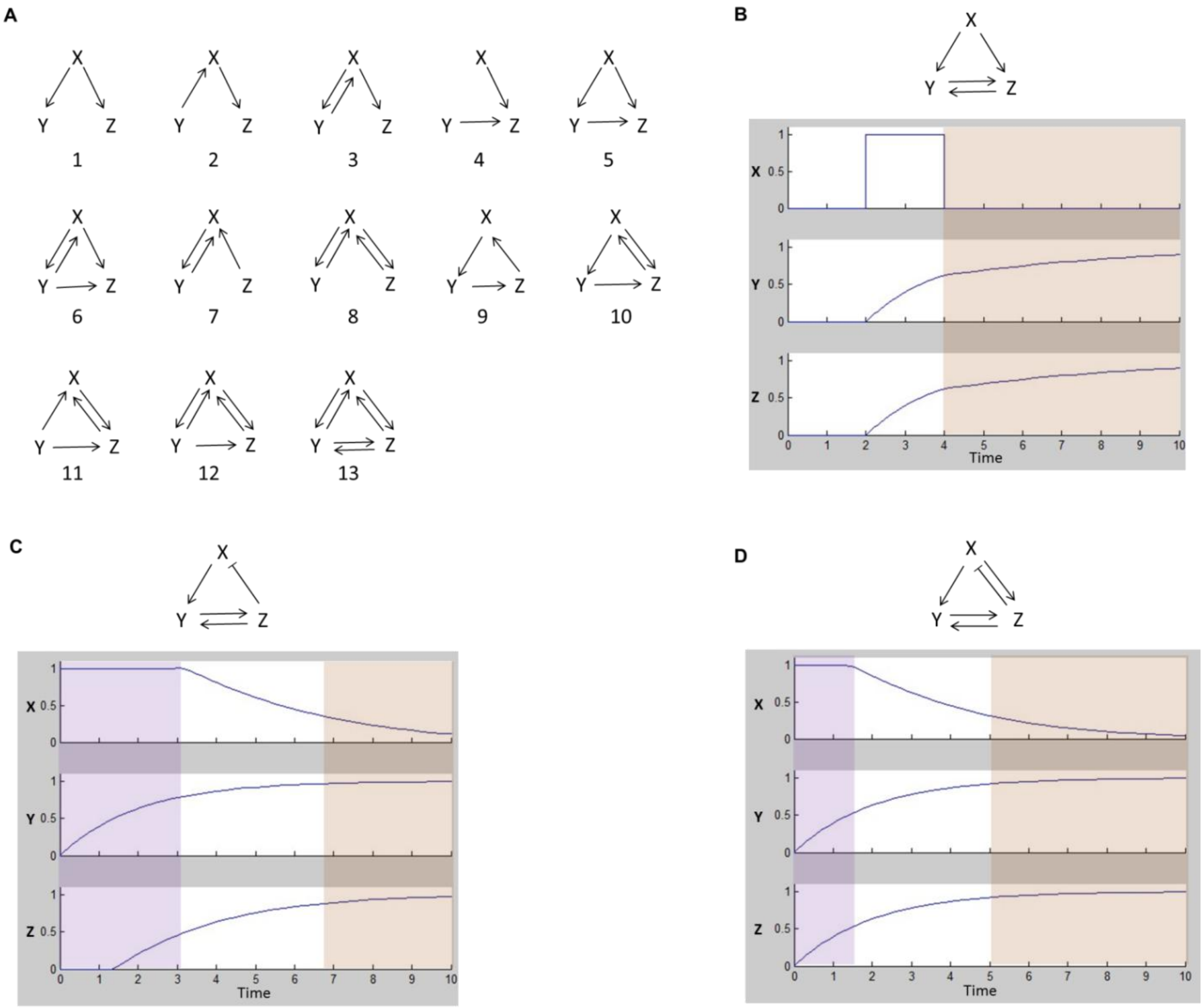

Supplementary Fig. S6. (*A*) All 13 three-node regulatory patterns. (*B-D*) Kinetic simulations of the functions of the three novel network motifs absent from the unicellular organisms *E. coli* and *S. cerevisiae*. (*B-D*) are the simulations for one case each of motifs 11, 10, and 12, respectively. In panel (*B*), X represents the signal; in panels (*C*) and (*D*), high X expression represents one state, and high Z expression represents another state. Kinetic simulations demonstrate that the novel motifs can fulfill the functions of maintenance and the transition of developmental states required for cell differentiation and fate decision in multicellular development.

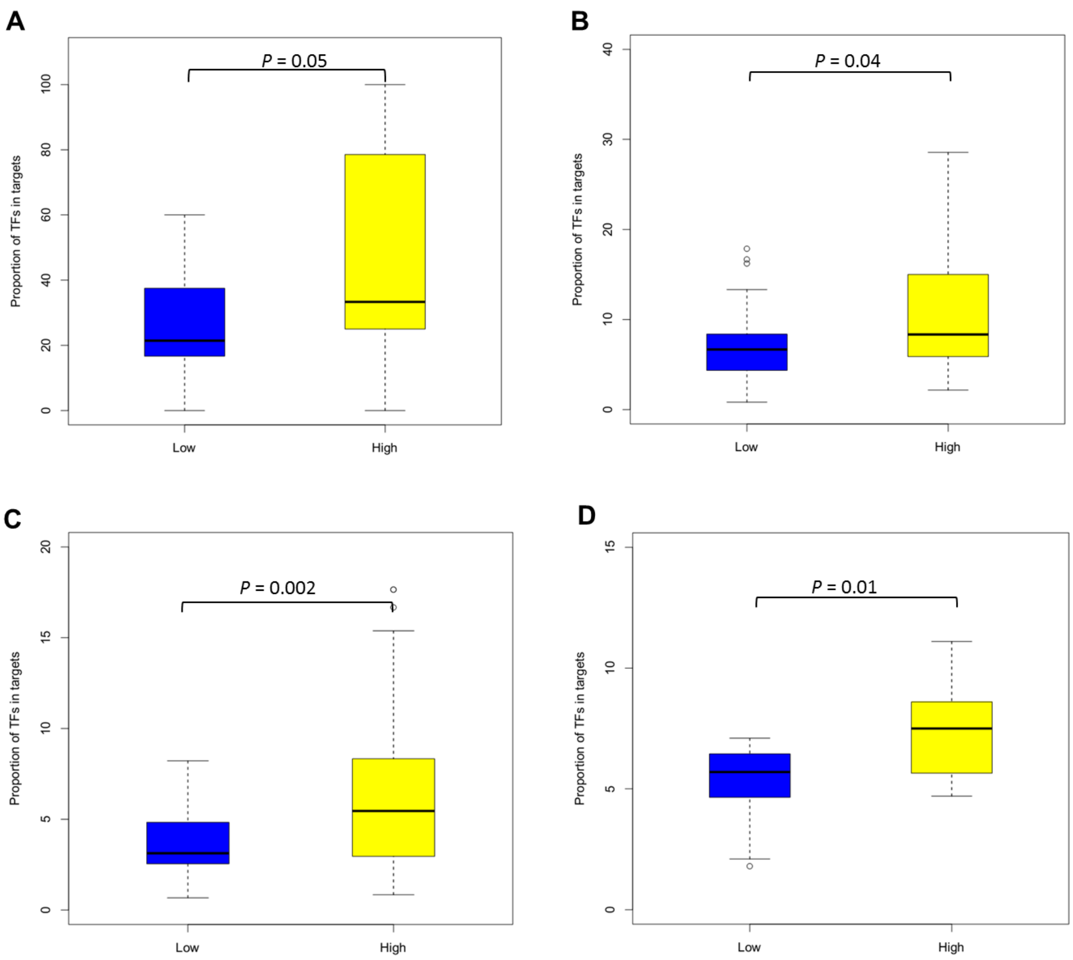

Supplementary Fig. S7. The binding specificities of TFs and the proportion of TFs to target genes in *A. thaliana* (*A*), *E. coli* (*B*), *S. cerevisiae* (*C*), and *H. sapiens* (*D*). One-tailed Wilcoxon rank-sum tests were performed between TFs with low and high binding specificities.

A

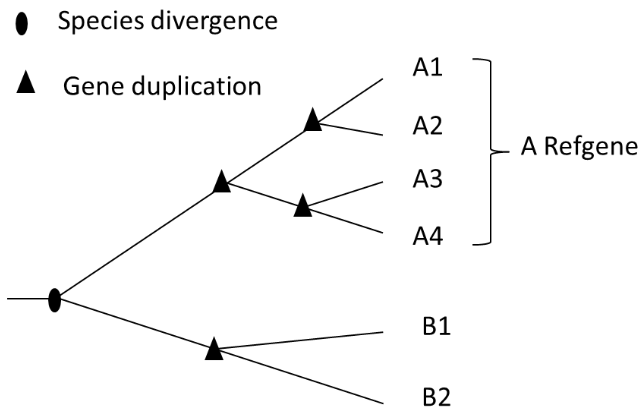

B

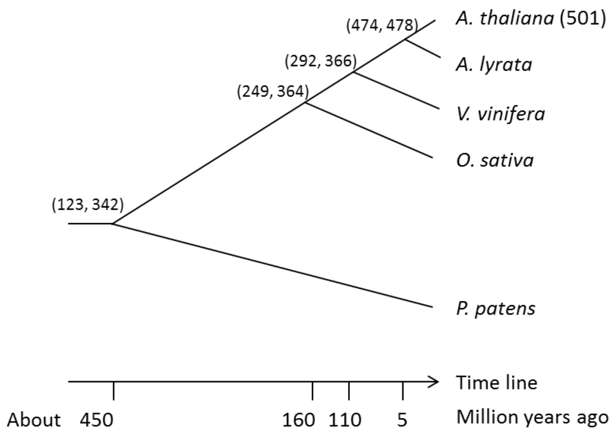

Supplementary Fig. S8. Clustering TFs descended from a common ancestor as a "Refgene" (*A*) and the four key time points used to cluster them (*B*). The numbers in parentheses represent the number of clusters and the number of TFs included in these clusters in *A. thaliana*.

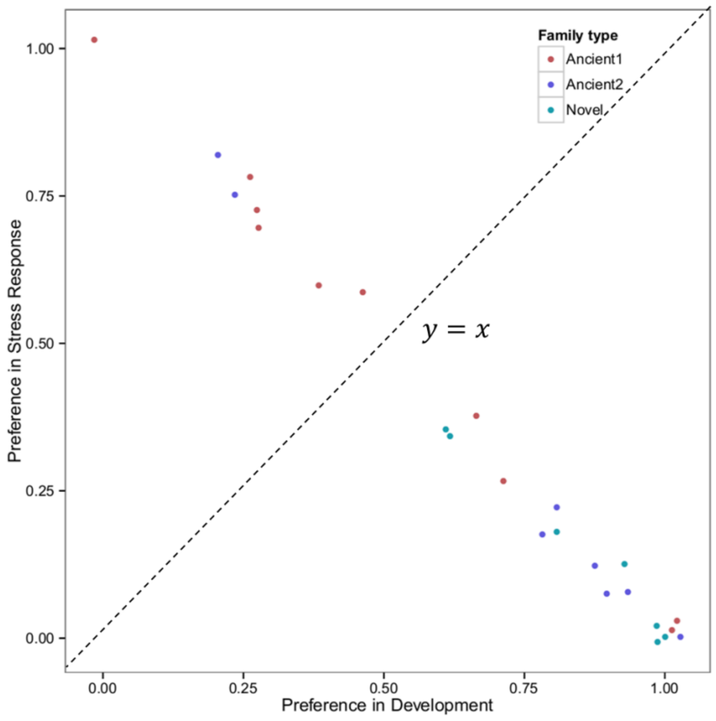

Supplementary Fig. S9. The wiring preferences of ancient and novel TF families in biological processes. Each point represents a family. Based on their presence in *E. coli*, *S. cerevisiae*, or *H. sapiens*, ancient families were further divided into two types, “Ancient1” and “Anicent2”. Ancient families present in any of the three species were classified as “Ancient1”, and those that were not present in the three species were classified as “Ancient2”. A jitter function was used to finely modify the point positions to display overlapping points.

Supplementary Tables:

Supplementary Table S1. Functional description of the 62 identified communities in the ATRM. For 62 of the identified communities containing no fewer than five members, GO enrichment for each community was performed using topGO, and genes with "Biological Process" annotation were used as the background. The *P* values were adjusted for multiple tests using the method of Benjamini and Hochberg. Based on the enriched GO terms, we assigned a name for the community whose enriched terms are consistent in terms of biological processes. (Excel file)

Supplementary Table S2. Topological structures of the predicted developmental and stress response sub-networks in *A. thaliana*. We used Match to predict these networks in the upstream 1,000 bp of the gene transcription start site (TSS) using binding matrices from TRANSFAC. Regulations with binding sites of no fewer than two and an expression correlation coefficient value (Pearson correlation coefficients, PCCs) of no lower than 0.30, 0.35, and 0.40 were adopted as the putative regulations. The PCCs for *Arabidopsis* genes were downloaded from ATTED-II (Obayashi, et al. 2009).

**PCC Threshold**	**Development**		**Stress response**	
	<Targets per TF>	TF-TF (%)[*]	<Targets per TF>	TF-TF (%)
**0.30**	23.5	28.9	37.4	21.6
**0.35**	13.6	36.1	26.5	23.0
**0.40**	6.8	40.7	18.1	26.5

[*]TF-TF (%): Proportion of regulations between two TFs

Supplementary Table S3. Three-node regulatory patterns screened from corresponding transcriptional regulatory networks using Mfinder 1.2. The identified network motifs are highlighted in blue under default thresholds (*P* < 0.01, Mfactor > 1.10 and Uniqueness ≥ 4) (Milo, et al. 2002).

Species	Motif ID	N_{real}	N_{Random} ± SD	*P*[*]	Uniqueness[$]
	1	281,705	282,482.2 ± 56.3	1.00	144
	2	2,561	3,329.8 ± 56.0	1.00	28
	3	1,105	1,467.5 ± 61.3	1.00	6
	4	3,384	4,333.1 ± 63.4	1.00	36
	5	1,145	376.4 ± 56.0	0	23
	6	230	49.6 ± 30.8	0	6
E. coli	7	36	54.6 ± 3.7	1.00	4
	8	0	3.6 ± 0.7	1.00	0
	9	0	0.3 ± 0.5	1.00	0
	10	2	1.0 ± 1.0	0.27	1
	11	12	3.5 ± 1.8	0	2
	12	1	0.4 ± 0.6	0.35	1
	13	1	0.0 ± 0.1	0.01	1
	1	89,808	89,897.8 ±10.4	1.00	86
	2	1,202	1,291.8 ±10.3	1.00	21
	3	271	271.9 ±4.1	0.69	2
	4	1,581	1,671.6 ±10.5	1.00	39
	5	147	57.0 ±10.3	0	12
	6	4	3.5 ±2.0	0.44	2
S. cerevisiae	7	11	10.3 ±1.0	0.64	2
	8	1	0.9 ±0.3	0.93	1
	9	0	0.0 ±0.2	1.00	0
	10	0	0.1 ±0.3	1.00	0
	11	0	0.2 ±0.5	1.00	0
	12	0	0.1 ±0.3	1.00	0
	13	0	0.0 ±0.0	1.00	0
	1	4,944	5,230.2 ± 7.9	1.00	109
A. thaliana	2	2,058	2,355.1 ± 9.0	1.00	52
	3	439	577.3 ± 4.9	1.00	17
	4	1,763	2,068.3 ± 7.7	1.00	77

5	303	46.2 ± 7.4	0	37
6	53	4.5 ± 2.1	0	10
7	286	386.0 ± 5.3	1.00	15
8	44	70.9 ± 2.5	1.00	6
9	9	1.9 ± 1.4	0.001	3
10	22	3.2 ± 1.9	0	6
11	34	4.6 ± 2.3	0	8
12	25	2.6 ± 1.6	0	8
13	2	0.5 ± 0.7	0.08	1

*P was calculated based on 1,000 randomized networks

$The number of distinct sets of nodes involved in this regulatory pattern in the real network

Supplementary Table S4. (*A*-*B*) Enriched biological processes (top 20) for TFs involved in the network motifs (motifs 5 and 6) that were also enriched in the unicellular organisms *E. coli* and *S. cerevisiae* (*A*) and enriched processes for those involved in the novel network motifs (motifs 10, 11, and 12) (*B*). The TFs with biological process annotations were used as the background. (*C*) Enriched biological processes ($P < 0.01$) for TFs involved in motif 10, which was absent from metazoan transcriptional regulatory networks. TFs involved in developmental processes were used as background to further explore the distribution of these motifs in developmental processes.

A

GO ID	GO term	*P*	Adjusted *P*
GO:0051093	negative regulation of developmental process	4.0e-07	0.0005
GO:0048518	positive regulation of biological process	2.1e-06	0.0013
GO:0031323	regulation of cellular metabolic process	4.8e-06	0.0017
GO:0019222	regulation of metabolic process	6.0e-06	0.0017
GO:0050794	regulation of cellular process	9.8e-06	0.0017
GO:0009893	positive regulation of metabolic process	1.0e-05	0.0017
GO:0031325	positive regulation of cellular metabolic process	1.0e-05	0.0017
GO:0048522	positive regulation of cellular process	1.6e-05	0.0021
GO:0044237	cellular metabolic process	1.8e-05	0.0021
GO:0065007	biological regulation	2.0e-05	0.0021
GO:0001708	cell fate specification	2.1e-05	0.0021
GO:0032501	multicellular organismal process	2.1e-05	0.0021
GO:0009987	cellular process	2.4e-05	0.0021
GO:0048856	anatomical structure development	2.5e-05	0.0021
GO:0050789	regulation of biological process	2.6e-05	0.0021
GO:0080090	regulation of primary metabolic process	3.9e-05	0.0026
GO:0010077	maintenance of inflorescence meristem identity	4.0e-05	0.0026
GO:0010187	negative regulation of seed germination	4.2e-05	0.0026
GO:0008152	metabolic process	4.2e-05	0.0026
GO:0048646	anatomical structure formation involved in morphogenesis	4.6e-05	0.0027

B

GO ID	GO term	*P*	Adjusted *P*
GO:0048731	system development	3.2e-16	3.8e-13
GO:0048856	anatomical structure development	7.9e-16	4.7e-13
GO:0007275	multicellular organismal development	3.6e-15	1.4e-12
GO:0032501	multicellular organismal process	7.9e-15	2.4e-12

GO:0032502	developmental process	4.2e-14	1.0e-11
GO:0048608	reproductive structure development	1.8e-13	3.6e-11
GO:0003006	developmental process involved in reproduction	9.6e-13	1.6e-10
GO:0000003	reproduction	2.1e-12	2.8e-10
GO:0022414	reproductive process	2.1e-12	2.8e-10
GO:0009888	tissue development	7.5e-12	8.9e-10
GO:0010073	meristem maintenance	3.4e-11	3.7e-09
GO:0007389	pattern specification process	9.6e-11	9.5e-09
GO:0003002	regionalization	1.1e-10	1.0e-08
GO:0048513	organ development	1.3e-10	1.1e-08
GO:0048507	meristem development	7.6e-10	6.0e-08
GO:0030154	cell differentiation	3.9e-09	2.9e-07
GO:0001708	cell fate specification	5.0e-09	3.4e-07
GO:0045165	cell fate commitment	5.1e-09	3.4e-07
GO:0009908	flower development	6.5e-09	4.1e-07
GO:0045596	negative regulation of cell differentiation	9.1e-09	5.3e-07

C

**GO ID**	**GO term**	***P***	**Adjusted *P***
GO:0010022	meristem determinacy	3.4e-09	1.8e-06
GO:0010582	floral meristem determinacy	3.4e-09	1.8e-06
GO:0010073	meristem maintenance	2.7e-08	9.3e-06
GO:0009888	tissue development	1.1e-07	2.5e-05
GO:0045596	negative regulation of cell differentiation	1.2e-07	2.5e-05
GO:0048513	organ development	5.7e-07	9.8e-05
GO:0048507	meristem development	1.0e-06	1.5e-04
GO:0010074	maintenance of meristem identity	2.3e-06	3.0e-04
GO:0045595	regulation of cell differentiation	5.7e-06	6.5e-04
GO:0019827	stem cell maintenance	8.0e-06	6.9e-04
GO:0048863	stem cell differentiation	8.0e-06	6.9e-04
GO:0048864	stem cell development	8.0e-06	6.9e-04
GO:0048523	negative regulation of cellular process	4.4e-05	0.0035
GO:0048468	cell development	5.2e-05	0.0038
GO:0030154	cell differentiation	6.8e-05	0.0047

Supplementary Table S5. TF numbers in each family in 28 species with genome sequences in PlantTFDB 2.0. By dating the time of emergence based on 28 plants with sequenced genomes, we classified 54 TF families present in the most recent common ancestor (MRCA) of land plants into two types: ancient and novel families. The TF families present in any of the nine green alga species were defined as ancient families (in black), and the TF families present in the MRCA of 19 land plants but absent from the nine green alga species were defined as novel families (in cyan). (Excel file)

Supplementary Table S6. (*A*) Origin types of the 19 novel TF families in *A. thaliana*. (*B*) Enriched biological processes (top 20) for the TFs of novel families. The TFs with biological process annotations with experimental evidence were used as the background.

A

**Origin type**	**Family**
New signature domain	BES1, EIL, GRAS, GRF, HRT-like, LBD, LFY, NAC, NZZ/SPL, SRS, STAT, TCP, Trihelix, VOZ, ZF-HD
New combination	ARF, HD-ZIP, MIKC, RAV

B

**GO ID**	**GO term**	***P***	**Adjusted *P***
GO:0048731	system development	3.9e-14	3.6e-11
GO:0048856	anatomical structure development	5.8e-14	3.6e-11
GO:0048513	organ development	1.3e-12	5.4e-10
GO:0032502	developmental process	1.8e-11	4.5e-09
GO:0007275	multicellular organismal development	1.8e-11	4.5e-09
GO:0032501	multicellular organismal process	2.4e-11	5.0e-09
GO:0022621	shoot system development	1.4e-10	2.2e-08
GO:0048367	shoot development	1.4e-10	2.2e-08
GO:0048366	leaf development	5.5e-09	7.6e-07
GO:0048827	phyllome development	1.3e-08	1.6e-06
GO:0010016	shoot morphogenesis	1.9e-08	2.1e-06
GO:0050793	regulation of developmental process	2.2e-07	2.3e-05
GO:0009965	leaf morphogenesis	1.3e-06	0.0001
GO:0051093	negative regulation of developmental process	2.8e-06	0.0003
GO:0009908	flower development	3.0e-06	0.0003
GO:0009791	post-embryonic development	5.9e-06	0.0005
GO:0009653	anatomical structure morphogenesis	8.8e-06	0.0006
GO:0048608	reproductive structure development	9.0e-06	0.0006
GO:0061458	reproductive system development	9.0e-06	0.0006
GO:0045962	positive regulation of development, heterochronic	1.8e-05	0.0011

Supplementary Table S7. Summary of ancient- and novel-family TFs in *E. coli*, *S. cerevisiae*, and *H. sapiens*.

**Species**		**Ancient**	**Novel**
E. coli	Family number	30	8
	TF number	216	9
	TF with regulatory specificity	64	1
S. cerevisiae	Family number	23	4
	TF number	104	62
	TF with regulatory specificity	56	21
H. Sapiens	Family number	32	24
	TF number	1,234	211
	TF with regulatory specificity	85	50

Supplementary Table S8. Correlation between the binding specificities of TFs and their wiring preferences in networks. (Excel file)

(*A*) Information content (IC) of TFs and their wiring in the ATRM. The degree is represented as the connectivity of this TF in the ATRM. The columns Motifs (5, 6) and Motifs (10, 11, 12) list the number of motifs in which this TF is involved. Based on their ICs, TFs were divided into two types: high IC (> the median IC [7.67]) and low IC (< the median IC).

(*B*) Correlation between the binding specificity of TFs and the proportion of TFs to target genes in *A. thaliana*. Spearman's rank correlation between the information content of TFs and the proportion of TFs to target genes: $\rho = 0.46$ and $P = 0.02$.

(*C*) Correlation between the binding specificity of TFs and the proportion of TFs to target genes in *E. coli*. Spearman's rank correlation between the information content of TFs and the proportion of TFs to target genes: $\rho = 0.31$ and $P = 0.03$.

(*D*) Correlation between the target number of TFs and the proportion of TFs to target genes in *S. cerevisiae*. Spearman's rank correlation between the TF target gene number and the proportion of TFs to target genes: $\rho = -0.36$ and $P = 0.0003$.

(*E*) Correlation between TF binding specificity and the proportion of TFs to target genes in *H. sapiens*. Spearman's rank correlation between the information content of TFs and the proportion of TFs to target genes: $\rho = 0.47$ and $P = 0.009$.

Supplementary Table S9. The wiring positions of novel- and ancient-family TFs in biological processes. The numbers in the table represent the number of TFs either without clustering (*A*) or with clustering based on the orthologous genes in *A. lyrata* (*B*), *V. vinifera* (*C*), *O. sativa* (*D*), and *P. patens* (*E*).

A

Type	Development	Stress response
Novel	109	24
Ancient	187	181

One-tailed Fisher's exact test: *P* = 8.69e-11, odds ratio= 4.38

B

Type	Development	Stress response
Novel	98	24
Ancient	179	173

One-tailed Fisher's exact test: *P* = 4.03e-09, odds ratio= 3.94

C

Type	Development	Stress response
Novel	70	17
Ancient	106	100

One-tailed Fisher's exact test: *P* = 1.79e-06, odds ratio= 3.87

D

Type	Development	Stress response
Novel	54	9
Ancient	95	91

One-tailed Fisher's exact test: *P* = 4.27e-07, odds ratio= 5.71

E

Type	Development	Stress response
Novel	26	7
Ancient	49	43

One-tailed Fisher's exact test: *P* = 0.008, odds ratio= 3.23

Supplementary Table S10. The wiring positions of novel- and ancient-family TFs that emerged during plant landing. The numbers in the table represent the number of TFs either without clustering (*A*) or with clustering based on the orthologous genes in *P. patens* (*B*).

A

Type	Development	Stress response
Novel	77	19
Ancient	80	81

One-tailed Fisher's exact test: *P* = 6.64e-07, odds ratio= 4.08

B

Type	Development	Stress response
Novel	25	6
Ancient	34	32

One-tailed Fisher's exact test: *P* = 0.007, odds ratio= 3.87

Supplementary Table S11. The wiring positions of young and old TFs in biological processes. We classified TFs emerged before the divergence of the MRCA of *A. thaliana* and *P. patens* as old TFs and classified those emerged after the divergence of the MRCA of *A. thaliana* and *P. patens* as young TFs.

**Type**	**Development**	**Stress response**
Young	123	95
Old	76	46

One-tailed Fisher's exact test: *P* = 0.87, odds ratio= 0.78

Supplementary References

Abdulrehman D, Monteiro PT, Teixeira MC, Mira NP, Lourenco AB, dos Santos SC, Cabrito TR, Francisco AP, Madeira SC, Aires RS, et al. 2011. YEASTRACT: providing a programmatic access to curated transcriptional regulatory associations in *Saccharomyces cerevisiae* through a web services interface. Nucleic Acids Res. 39:D136-140.

Alexa A, Rahnenfuhrer J. 2010. topGO: Enrichment analysis for Gene Ontology. R package version 2.10.0. http://www.bioconductor.org/packages/release/bioc/html/topGO.html.

Altschul SF, Madden TL, Schaffer AA, Zhang J, Zhang Z, Miller W, Lipman DJ. 1997. Gapped BLAST and PSI-BLAST: a new generation of protein database search programs. Nucleic Acids Res. 25:3389-3402.

Benjamini Y, Hochberg Y. 1995. Controlling the false discovery rate: a practical and powerful approach to multiple testing. Journal of the Royal Statistical Society. Series B (Methodological):289-300.

Berardini TZ, Mundodi S, Reiser L, Huala E, Garcia-Hernandez M, Zhang P, Mueller LA, Yoon J, Doyle A, Lander G. 2004. Functional annotation of the *Arabidopsis* genome using controlled vocabularies. Plant physiology 135:745-755.

Charoensawan V, Wilson D, Teichmann SA. 2010. Lineage-specific expansion of DNA-binding transcription factor families. Trends Genet. 26:388-393.

Csardi G, Nepusz T. 2006. The igraph software package for complex network research. InterJournal, Complex Systems 1695:38.

Cutler SR, Rodriguez PL, Finkelstein RR, Abrams SR. 2010. Abscisic acid: emergence of a core signaling network. Annu Rev Plant Biol. 61:651-679.

Fortunato S. 2010. Community detection in graphs. Physics Reports 486:75-174.

Gerstein MB, Kundaje A, Hariharan M, Landt SG, Yan KK, Cheng C, Mu XJ, Khurana E, Rozowsky J, Alexander R, et al. 2012. Architecture of the human regulatory network derived from ENCODE data. Nature 489:91-100.

Guzzi PH, Cannataro M editors. Computer-Based Medical Systems (CBMS), 2012 25th International Symposium on. 2012.

Hertz GZ, Stormo GD. 1999. Identifying DNA and protein patterns with statistically significant alignments of multiple sequences. Bioinformatics 15:563-577.

Irish VF. 2010. The flowering of *Arabidopsis* flower development. Plant J. 61:1014-1028.

Kersey PJ, Staines DM, Lawson D, Kulesha E, Derwent P, Humphrey JC, Hughes DS, Keenan S, Kerhornou A, Koscielny G, et al. 2012. Ensembl Genomes: an integrative resource for genome-scale data from non-vertebrate species. Nucleic Acids Res. 40:D91-97.

Luscombe NM, Babu MM, Yu H, Snyder M, Teichmann SA, Gerstein M. 2004. Genomic analysis of regulatory network dynamics reveals large topological changes. Nature 431:308-312.

Mangan S, Alon U. 2003. Structure and function of the feed-forward loop network motif. Proc Natl Acad Sci U S A. 100:11980-11985.

Matys V, Kel-Margoulis OV, Fricke E, Liebich I, Land S, Barre-Dirrie A, Reuter I, Chekmenev D, Krull M, Hornischer K, et al. 2006. TRANSFAC and its module TRANSCompel: transcriptional gene regulation in eukaryotes. Nucleic Acids Res. 34:D108-110.

Milo R, Shen-Orr S, Itzkovitz S, Kashtan N, Chklovskii D, Alon U. 2002. Network motifs: simple building blocks of complex networks. Science 298:824-827.

Nikitin A, Egorov S, Daraselia N, Mazo I. 2003. Pathway studio--the analysis and navigation of molecular networks. Bioinformatics 19:2155-2157.

Novichkova S, Egorov S, Daraselia N. 2003. MedScan, a natural language processing engine for MEDLINE abstracts. Bioinformatics 19:1699-1706.

Obayashi T, Hayashi S, Saeki M, Ohta H, Kinoshita K. 2009. ATTED-II provides coexpressed gene networks for *Arabidopsis*. Nucleic Acids Res. 37:D987-991.

Salgado H, Peralta-Gil M, Gama-Castro S, Santos-Zavaleta A, Muniz-Rascado L, Garcia-Sotelo JS, Weiss V, Solano-Lira H, Martinez-Flores I, Medina-Rivera A, et al. 2013. RegulonDB v8.0: omics data sets, evolutionary conservation, regulatory phrases, cross-validated gold standards and more. Nucleic Acids Res. 41:D203-213.

Schneider TD, Stormo GD, Gold L, Ehrenfeucht A. 1986. Information content of binding sites on nucleotide sequences. J Mol Biol. 188:415-431.

Wilson D, Charoensawan V, Kummerfeld SK, Teichmann SA. 2008. DBD--taxonomically broad transcription factor predictions: new content and functionality. Nucleic Acids Res. 36:D88-92.

Yilmaz A, Mejia-Guerra MK, Kurz K, Liang X, Welch L, Grotewold E. 2011. AGRIS: the *Arabidopsis* Gene Regulatory Information Server, an update. Nucleic Acids Res. 39:D1118-1122.

Zhang H, Jin J, Tang L, Zhao Y, Gu X, Gao G, Luo J. 2011. PlantTFDB 2.0: update and improvement of the comprehensive plant transcription factor database. Nucleic Acids Res. 39:D1114-1117.
